# A Novel CMKLR1 Small Molecule Antagonist Suppresses CNS Autoimmune Inflammatory Disease

**DOI:** 10.1371/journal.pone.0112925

**Published:** 2014-12-01

**Authors:** Kareem L. Graham, Jian V. Zhang, Susanna Lewén, Thomas M. Burke, Ton Dang, Maria Zoudilova, Raymond A. Sobel, Eugene C. Butcher, Brian A. Zabel

**Affiliations:** 1 Palo Alto Veterans Institute for Research and Veterans Affairs Palo Alto Health Care System, Palo Alto, California, United States of America; 2 ChemoCentryx, Inc., Mountain View, California, United States of America; 3 Department of Pathology, Stanford University School of Medicine, Stanford, California, United States of America; University Hospital of Heidelberg, Germany

## Abstract

Therapies that target leukocyte trafficking pathways can reduce disease activity and improve clinical outcomes in multiple sclerosis (MS). Experimental autoimmune encephalomyelitis (EAE) is a widely studied animal model that shares many clinical and histological features with MS. Chemokine-like receptor-^1^ (CMKLR1) is a chemoattractant receptor that is expressed by key effector cells in EAE and MS, including macrophages, subsets of dendritic cells, natural killer cells and microglia. We previously showed that CMKLR1-deficient (CMKLR1 KO) mice develop less severe clinical and histological EAE than wild-type mice. In this study, we sought to identify CMKLR1 inhibitors that would pharmaceutically recapitulate the CMKLR1 KO phenotype in EAE. We identified 2-(α-naphthoyl) ethyltrimethylammonium iodide (α-NETA) as a CMKLR1 small molecule antagonist that inhibits chemerin-stimulated β-arrestin2 association with CMKLR1, as well as chemerin-triggered CMKLR1^+^ cell migration. α-NETA significantly delayed the onset of EAE induced in C57BL/6 mice by both active immunization with myelin oligodendrocyte glycoprotein peptide 35-55 and by adoptive transfer of encephalitogenic T cells. In addition, α-NETA treatment significantly reduced mononuclear cell infiltrates within the CNS. This study provides additional proof-of-concept data that targeting CMKLR1:chemerin interactions may be beneficial in preventing or treating MS.

## Introduction

Multiple sclerosis (MS) is a demyelinating disease of the CNS that affects approximately 2 million people worldwide. Tissue injury in MS and experimental autoimmune encephalomyelitis (EAE), its widely studied animal model, is mediated in part by inflammatory leukocytes that transmigrate across the blood-brain barrier [1]. Therapies that target leukocyte trafficking pathways can reduce disease activity and improve clinical outcomes in MS. Currently approved disease-modifying drugs for MS that function by altering systemic leukocyte migration or distribution (e.g., Tysabri, an anti-α4 integrin adhesion molecule antibody, or Gilenya, a small molecule sphingosine-1-phosphate receptor modulator) are, however, associated with potentially severe side effects in some patients [2], [3], [4], [5], [6], [7]. Agents that selectively target the trafficking of key inflammatory cell subsets involved in the pathophysiology of MS may therefore be superior to current treatment strategies.

Chemokine-like receptor-1 (CMKLR1) is G protein-coupled receptor (GPCR) that binds chemerin, a proteolytically regulated leukocyte chemoattractant. CMKLR1 protein is expressed by macrophages, subsets of dendritic cells, natural killer (NK) cells and microglia [8], [9], [10], [11], [12]. There are several lines of evidence that point to pivotal roles for CMKLR1 in pathogenic CNS inflammation. For one, CMKLR1-knockout (KO) mice develop less severe clinical and histological EAE than wild-type (WT) mice [12]. In addition, chemerin co-localizes with intralesional endothelial cells in the brains of MS patients, and CMKLR1^+^ dendritic cells are present in the leptomeninges and in perivascular cuffs of chronic and active MS lesions [13]. CMKLR1 may therefore represent a novel target for the treatment of MS. Suitable pharmaco-inhibitors of CMKLR1, however, remain to be identified and tested in models of autoimmune CNS inflammation. In this study, we used a functional whole-cell assay to screen for novel small molecule inhibitors of CMKLR1 activity, with the goal of identifying lead compounds for evaluation in the EAE model of MS.

## Materials and Methods

### Ethics statement

All animal studies and procedures were approved by the Institutional Animal Use and Care Committee at the Veterans Affairs Palo Alto Health Care System (animal welfare assurance number A3088-01; AAALAC-accredited facility).

### Mice and reagents

C57BL/6 mice were purchased from The Jackson Laboratory, and female mice (8–12 weeks old) were used in all experiments. CMKLR1 knockout (KO) mice were obtained from Deltagen and fully backcrossed (nine generations) onto the C57BL/6 background [12]. CCRL2 KO mice were obtained from Jackson Labs fully backcrossed on the C57BL/6 background [14]. α-NETA (purchased from ENZO, Santa Cruz, CA and Proactive Molecular Research, Alachua, FL) was formulated in 10% captisol vehicle (Cydex Pharmaceuticals) for *in vivo* dosing. All animal experiments were conducted in accordance with approved Veterans Affairs, National Institutes of Health, and Institutional Animal Care and Use Committee guidelines. Myelin oligodendrocyte glycoprotein (MOG) peptide amino acids 35–35 (MEVGWYRSPFSRVVHLYRNGK; MOG_35–55_) was synthesized by the Stanford Protein and Nucleic Acid Facility (Stanford, CA). Complete Freund's adjuvant (CFA) consisted of incomplete Freund's adjuvant (Difco) plus 4 mg/ml heat-inactivated *Mycobacterium tuberculosis* (strain H37 RA; Difco).

### β-arrestin2 (β-ARR2) assay and compound library screen

The compound library screen was performed at the Stanford High Throughput Bioscience Center (HTBC). The Stanford HTBC compound library contains ∼130,000 diverse compounds from ChemDiv (60,000), SPECS (30,000), Chembridge (23,500), ChemRX (10,000), Microsource Sepctrum,(2,320), Enzo ICCB Known Bioactives (472) and FDA Approved Drug Library (780), library of pharmaceutically active compounds (LOPAC) (1,280), NIH Clinical Collection (727), and NCI Developmental Therapeutics Program Approved Oncology Drugs (114). A total of 3×10^3^ CHO-CMKLR1-βgal1:β-arrestin2-βgal2 (CMKLR1/CHO) cells (DiscoveRx) in 30 µl media were seeded into each well of 384-well plates and cultured overnight. Small molecule compounds were then added to each well (0.1 µl of 10 mM stock compound in DMSO; 25 µM final compound concentration). Chemerin agonist was then added (10 µl; 10 nM final concentration, R&D Systems). After 60 min, 20 µl of chemiluminescent substrate (DiscoveRx) was added, and 1 h later luminescent signal detected. For the 96-well plate assays, 2×10^4^ CMKLR1/CHO, CXCR7/CHO), or GPR1/CHO β-arrestin cell lines (DiscoveRx) were seeded into 96-well plates and cultured overnight. The next day, the medium was removed by aspiration, 100 µl of 0.1% DMSO (in PBS) containing small molecule compounds was added to the wells, and the plates were pre-incubated at room temperature. After 10 minutes, 5 µl of chemoattractant agonist was added, and the plates were incubated in 5% CO_2_ at 37°C. After 90 min, 50 µl of chemilluminescent substrate (DiscoveRx) was added to the wells and the plates were incubated at room temperature. After 1 h, light emission was analyzed in a Top-Count scintillation counter (Perkin Elmer). α-NEDA, naphthalene, and PTEG tested in this assay were purchased from Proactive Molecular Research.

### Chemotaxis assays

Transwell assay plates were used to evaluate chemotaxis *in vitro* [15]. Briefly, mouse (m) and human (hu) CMKLR1 transfectants (generated in mouse L1.2 pre-B cell lymphoma cells), MOLT-4, U937, or NC-37 cells were pre-treated for 10 min at 37°C with DMSO vehicle control or various concentrations of α-NETA and in some cases α-NEDA or naphthalene. We then assessed CMKLR1/L1.2 cell migration to 1 nM chemerin, CCR9+ MOLT-4 cell migration to 250 nM CCL25, CXCR4+ U937 cell migration to 100 nM CXCL12, and CXCR5+ NC-37 cell migration to150 nM CXCL13 (chemoattractants from R&D Systems) using 5 µm pore transwell chemotaxis chambers (Costar). Cells were allowed to migrate for 90 min at 37°C, 8% CO_2_ and then quantified by flow cytometry.

### Compound stability in mouse plasma

α-NETA and Benfluorex were initially solubilized in acetonitrile:water (1∶1) at 2 mM, and then diluted to either 10 µM (α-NETA) or 1 µM (Benfluorex) in normal mouse plasma (C56BL/6 origin), with a final volume of 0.8 ml. The samples were incubated at the indicated times and temperatures, quenched with 0.2 ml of 200 ng/ml IS (Phenacetin) in 0.1% FA/Acetonitrile:Methanol (90∶10), and analyzed on an API3000 (System 2) LC/MS using a C18 column (0.45 ml/min flow rate).

### EAE induction

Mice were immunized via subcutaneous (s.c.) injection with 100 µg MOG_35–55_ emulsified in CFA on day 0. Mice also received 250 ng Pertussis toxin (List Biological Labs) i.v. via tail vein injection at the time of MOG_35–55_/CFA immunization and again 2 days later. Beginning on the day of MOG_35–55_/CFA immunization, mice received α-NETA or 10% captisol vehicle via s.c. injection (100 µl volume). Mice continued to receive α-NETA or captisol daily until the end of the experiment. Clinical disease was scored as follows: 0 =  normal or healthy; 1 =  flaccid tail; 2 =  hindlimb weakness; 3 =  hindlimb paralysis; 4 =  hindlimb and forelimb paralysis; 5 =  moribund or dead. Individuals who conducted the clinical scoring (K.L.G, M.Z. or B.A.Z.) were blinded to the treatment that the mice received. For induction of EAE by adoptive transfer, female, C57BL/6 mice (8–10 weeks of age) were immunized s.c. with 100 µg MOG_35–55_ in CFA. Beginning at the time of immunization, mice received once daily injections of α-NETA or 10% captisol. Ten days after immunization, we harvested draining lymph node (LN) and spleen cells. We then resuspended cells in RPMI 1640 media with supplements, followed by incubation at 5×10^6^ cells/ml with MOG_35–55_ (10 µg/ml) and recombinant murine IL-12 (10 ng/ml; R&D Systems). After 4 days in culture (37°C, 8% CO_2_), lymphocytes were isolated using Lympholyte-Mammal (Cedarlane Laboratories). Female, C57BL/6 mice (10 weeks of age) received 1–2×10^7^ viable MOG-reactive cells derived from α-NETA- or vehicle-treated mice via i.v. injection. Pertussis toxin (400 ng) was given i.v. immediately after cell transfer and again 2 days later. Where indicated, mice received once daily injections of α-NETA or 10% captisol beginning at the time of cell transfer.

### ELISA

Mice were immunized s.c. with 100 µg MOG_35–55_ in CFA. Beginning at the time of immunization, mice received daily injections of α-NETA or 10% captisol. Ten days after initial immunization, draining LN and spleen cells were harvested, resuspended in RPMI 1640 with supplements, and then incubated at 2×10^5^ cells/well (37°C, 8% CO_2_) in the presence of a dose-range of MOG_35–55_. Culture supernatants were harvested after 72 h, and cytokine levels in triplicate wells were determined by sandwich ELISA according to the manufacturer's instructions (BD Pharmingen, eBioscience).

### Proliferation assays

LN and spleen cells were cultured as described above for 72 h in triplicate wells with a range of MOG_35–55_ concentrations. We then added [^3^H] Thymidine (Perkin Elmer) for the last 18–24 h of culture and assessed thymidine incorporation using a β-plate scintillation counter.

### CNS mononuclear cell preparation

Mice were perfused through the heart with 30 ml cold PBS. We then extracted spinal cords and brain stems, which were minced and incubated with HBSS containing 0.4 U per cord/stem of Liberase TL (Roche), 50 µg/ml DNase I (Roche), and 25 mM HEPES for 45 min at 37°C. Digested tissue was forced through stainless steel mesh and mononuclear cells were collected from 30∶70% discontinuous Percoll gradients.

### Flow cytometry

MAbs directed against mouse CD3 (145–2C11), CD11b (M1/70), CD11c (N418), F4/80 (BM8), B220 (RA3-6B2) and NK1.1 (PK136) were from eBioscience or BD Pharmingen. Staining buffer consisted of PBS containing 2% BSA plus 0.1% sodium azide.

### Histology

Brain and spinal cord tissues were fixed in 10% buffered formalin. Paraffin-embedded tissues were then stained with Luxol fast blue-H&E stain, and CNS inflammatory foci (>10 mononuclear cells/focus) in leptomeninges and parenchyma were counted in each mouse sample in a blinded fashion by one of the authors (R.A.S.).

### Statistics

The Mann-Whitney *U* test was applied to analyze nonparametric clinical EAE data, and the Student's *t* test was used to analyze all parametric data. The Fisher's exact test was used to compare disease incidence. Values of *p* less than 0.05 were considered statistically significant.

## Results

### α-NETA is a novel inhibitor of chemerin-stimulated β-arrestin2 association with CMKLR1

**Figure 1:**
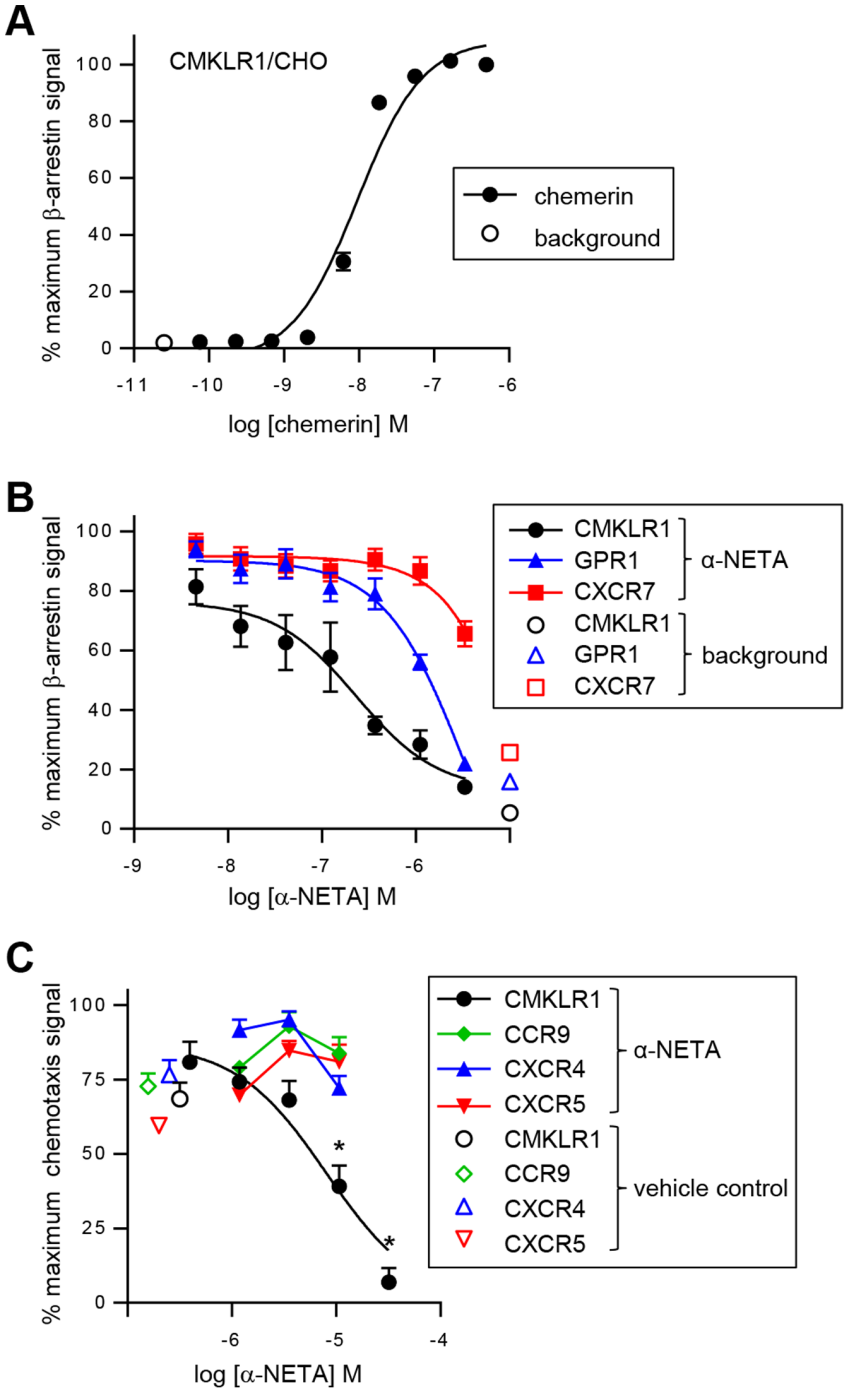
α-NETA selectively inhibits chemerin-stimulated CMKLR1 association with β-arrestin2. (A) Chemerin triggered the association of β-arrestin2 with CMKLR1 in a concentration-dependent manner, as measured by enzymatic complementation assay and quantified by chemiluminescence. For each point the mean ± SEM of n = 9 independent experiments is shown. (B) α-NETA was tested for potency in inhibiting chemerin-mediated β-arrestin2 association with CMKLR1. CMKLR1/β-ARR2 CHO cells were incubated with the indicated concentrations of α-NETA and 7 nM recombinant chemerin (background signal was evaluated in the absence of α-NETA and chemerin). α-NETA was tested for selectivity by evaluating inhibition of chemerin-mediated β-arrestin2 association with GPR1 or CXCL12-mediated β-arrestin2 association with CXCR7. For each point the mean ± SEM of n = 18-19 independent experiments is shown for CMKLR1 and CXCR7, and the mean ± SEM of triplicate wells for one experiment is shown for GPR1. (C) α-NETA was tested for potency and selectivity in inhibiting chemerin-mediated CMKLR1+ cell migration. CMKLR1+ L1.2 cells, CCR9+ MOLT4 cells, CXCR4+ U937 cells, and CXCR5+ NC-37 cells were incubated with the indicated concentrations of α-NETA and then tested for transwell migration to their cognate attractant ligands (7 nM chemerin, 250 nM CCL25, 100 nM CXCL12, 150 nM CXCL13, respectively). Chemotaxis for each cell type was normalized to its maximum migration signal, and cell migration at each concentration of α-NETA was compared with vehicle (0.1% DMSO) for inhibition. For each point the mean ± SEM of 3–5 wells from n = 1–3 independent experiments is shown. *p<0.05 by Student's t-test.

The engagement of a chemoattractant receptor by its cognate ligand stimulates the recruitment of β-arrestin to the carboxyl-terminus of the receptor, an event that results in receptor internalization and termination of G protein-mediated signaling [16]. We previously showed that chemerin binding to CMKLR1 induced rapid and efficient receptor internalization [17], and thus reasoned that chemerin likely triggers association of β-arrestins with CMKLR1. To test this, we used an enzymatic complementation assay (the β-ARR2 assay), whereby the complementary fragments from the β-gal active site are fused to the carboxyl-terminus of CMKLR1 and β-arrestin2. The special association of these molecules leads to enzyme activity, which is detected by a chemiluminescent substrate. As anticipated, chemerin triggered robust association of CMKLR1 with β-arrestin2, with an EC_50_ value of 9.7±0.7 nM (n = 9, mean ± SEM; Fig. 1A).

We used the β-ARR2 assay to screen a small molecule library for novel modulators of CMKLR1 activity. The compounds were tested at 10–25 µM, and a ‘hit’ was defined as >40% inhibition. We used the well-known β-galactosidase small molecule inhibitor phenylethyl β-d-thiogalactoside (PTEG) as a positive control. From this screen, we identified 2-(α-naphthoyl)ethyltrimethylammonium iodide (α-NETA) as a potent inhibitor of chemerin-stimulated β-ARR2 association with CMKLR1, IC_50_: 375±42 nM (n = 14, mean ± SEM, Fig. 1B).

### α-NETA target selectivity *in vitro*

α-NETA was originally identified as an inhibitor of choline acetyltransferase (ChAT, IC_50_: 9 µM) [18], an enzyme that catalyzes the synthesis of acetylcholine from choline and acetyl-CoA. α-NETA had substantially reduced potency against cholinesterase (IC_50_: 84 µM) and acetylcholinesterase (IC_50_:300 µM) [19] (Table 1). Compared to its effects on ChAT activity, α-NETA was a more potent inhibitor of chemerin:CMKLR1 signaling (IC_50_: 375 nM for β-ARR2 recruitment) (Fig. 1, Table 1). Since its discovery as a ChAT inhibitor, α-NETA has been screened against at least 102 discrete targets ranging from enzymes (e.g. aldehyde dehydrogenase 1 family, member A1 (ALDH1A1), Cruzain) to processes (e.g. viral binding/entry, DNA re-replication) to GPCRs (e.g. GPR154, prostaglandin E2 receptor) (Table 1) [20], [21]. α -NETA was inactive against carnitine acetyletransferase [19] as well as 55 additional targets, with inconclusive results against 24 (inconclusive defined as partial inhibition, partial curve, or discrepant data [20]). α-NETA was most potent in inhibiting ALDH1A1 (IC_50_: 0.04 µM) [22], followed by CMKLR1 (IC_50_: 0.38 µM) and G9a histone lysine methyltransferase (IC_50_: 0.50 µM) [23], with the caveat that ALDH1A1 and G9a were tested in isolation as purified enzymes (Table 1).

**Table 1:** α-NETA target selectivity.

Target	IC_50_ (µM)	Screening assay	AID^a^
Aldehyde dehydrogenase 1 1 family, member A1 (ALDH1A1)^b^	0.04	Purified enzyme	493210
Cytochrome p450 3A4^c^^,^ d	0.20	Purified enzyme	885
**CMKLR1** e	0.38	Cell-based (GPCR)	504332
G9a histone lysine methyltransferase^b^	0.50	Purified enzyme	686979
Tyrosyl-DNA phosphodiesterase 1 + camptothecin^c^	0.58	Cell-based (process)	444
Nuclear factor of activated T cells (NFAT)^c^	1.3	Cell-based	463097
DNA re-replication^b^	1.8^f^	Cell-based (process)	504847
Vitamin D receptor^c^	2.0	Purified protein (binding)	918
Thrombopoietin^c^	2.5	Cell-based	540276
GPR1^e^	3.4	Cell-based (GPCR)	446
Marburg virus binding/entry^b^	4.5	Cell-based	1851
IL-6/STAT signaling^c^	6.3	Cell-based	488949
Cytochrome p450 1A2^c^	8	Purified enzyme	1476
Choline acetyltransferase^g^	9	Purified enzyme	463096
MPP8 chromodomain/methylated histones^b^	10	Purified protein (binding)	1851
Cruzain^b^	10	Purified enzyme	1851
Lassa virus binding/entry^b^	10	Cell-based	924
CCR9^e^	≥10	Cell-based (GPCR)	894
CXCR4^e^	≥10	Cell-based (GPCR)	10839
CXCR5^e^	≥10	Cell-based (GPCR)	1422
CXCR7^e^	≥10	Cell-based (GPCR)	912
Cytochrome p450 2D6 c ^,^ d	≥10	Purified enzyme	1461
Cytochrome p450 2C9^c^^,^ d	13	Purified enzyme	588834
p53 mutants^c^	13	Cell-based (process)	1851
15-Hydroxyprostaglandin dehydrogenase^b^	14	Purified enzyme	943
*Bacillus subtilis* Sfp phosphopantetheinyl transferase^c^	18	Purified enzyme	493210
Prostaglandin E2 receptor^b^	n.d.^h^	Cell-based (GPCR)	885
*Bacillus anthracis* lethal toxin^c^	25	Cell-based (process)	504332
GPR154^b^	32	Cell-based (GPCR)	686979
Cholinesterase^g^	84	Purified enzyme	444
Acetylcholinesterase^g^	300	Purified enzyme	463097
hERG^b^	Inactive^i^	Cell-based	504847
Cytochrome p450 2C19^c^	Inactive^j^	Purified enzyme	918
Acetylcholine muscarinic M1 receptor^c^	Inactive^k^	Cell-based (GPCR)	540276

a PubChem BioAssay identifier.

b PubChem, 2014b.

c PubChem, 2014a.

d Partial effect; 46-58% maximum inhibition at up to 57 µM.

e This study.

f Agonist activity, EC_50_ (µM).

g Sastry et al., 1988a.

h Single dose (25 µM) tested, 106% inhibition.

i Tested up to 92 µM.

j Tested up to 57 µM.

k Tested up to 35 µM.

We next focused on assessing the selectivity of α-NETA for CMKLR1 compared with other GPCRs in cell-based assays. α-NETA was a poor inhibitor of neuropeptide S-induced GPR154 signaling (IC_50_: 32 µM) [24], and was inactive against acetylcholine muscarinic M1 receptor (tested up to 35 µM) [25] (Table 1). The compound completely inhibited prostaglandin E2-induced receptor signaling when tested at 25 µM, although potency data is unavailable as the compound was tested only at a single concentration [26] (Table 1). We directly tested the compound for inhibitory activity in ligand-triggered β-arrestin2 recruitment to related chemerin receptor GPR1, and to unrelated GPCR CXCR7. α-NETA inhibited chemerin-stimulated β-arrestin2 association with GPR1 (IC_50_: 3.4 µM), although the antagonist activity was 10-fold less potent compared to its activity against CMKLR1 (Fig. 1B, Table 1). While 3 µM α-NETA moderately inhibited CXCL12-triggered β-arrestin2 association with CXCR7, lower concentrations of α-NETA had no-to-minimal inhibitory activity (IC_50_: >10 µM), overall indicating some degree of selectivity for CMKLR1 (Fig. 1B, Table 1).

### α-NETA inhibits CMKLR1^+^ cell migration

Chemerin induces migration of CMKLR1^+^ cells [9], [27]. Thus, we sought to determine if α-NETA could modulate CMKLR1^+^ cell chemotaxis to chemerin. Indeed, α-NETA significantly inhibited chemerin-mediated CMKLR1^+^ cell migration in an *in vitro* transwell chemotaxis assay (IC_50_: 6.5±0.7 µM; Fig. 1C, Table 2). In additional selectivity testing, 1, 3, and 10 µM α-NETA did not inhibit the migration of CCR9+ MOLT4 cells to CCL25, CXCR4+ U394 cells to CXCL12, or CXCR5+ NC-37 cells to CXCL13 (Fig. 1C)(Table 1). Importantly, α-NETA inhibited migration of cells expressing mouse CMKLR1 with potency similar to cells expressing the human form of the receptor (Table 2). Thus, overall among the GPCRs tested or reported, α-NETA is relatively selective for CMKLR1.

**Table 2:** Structure-activity relationship: charged ethyltrimethylamine critical for target potency.

Assay	Target	α-NETA^a^	α-NEDA^a^	naphthalene^a^
β-arrestin	huCMKLR1	0.38±0.04	7.50±0.74	>10
chemotaxis	huCMKLR1	6.5±0.7	77±23	>100
chemotaxis	mCMKLR1	5.0±0.4	78±22	>100

a IC_50_ (µM), mean±SEM, n = 3-14 independent experiments per assay per compound.

### Structure-activity relationship

To identify structural features of α-NETA required for antagonist activity against CMKLR1, we evaluated the related compound 2-(α-naphthoyl)ethyldimethylamine (α-NEDA) as well as the simple bicyclic benzenoid naphthalene for inhibitory activity in β-ARR2 and transwell migration assays. Naphthalene had no inhibitory activity in the *in vitro* assays, and α-NEDA was 10–20-fold less potent than α-NETA in inhibiting chemerin-stimulated β-ARR2 association with CMKLR1 or CMKLR1+ cell migration (Table 2). These results indicate that the charged ethyltrimethylamine domain of the α-substituted ketone is critical for the inhibitory action of α-NETA against CMKLR1. Interestingly, Sastry et al. reported that the charged nitrogen also contributed to ChAT target potency, as α-NEDA was 7-fold less potent than α-NETA in inhibiting enzyme activity [19].

### α-NETA stability

**Figure 2:**
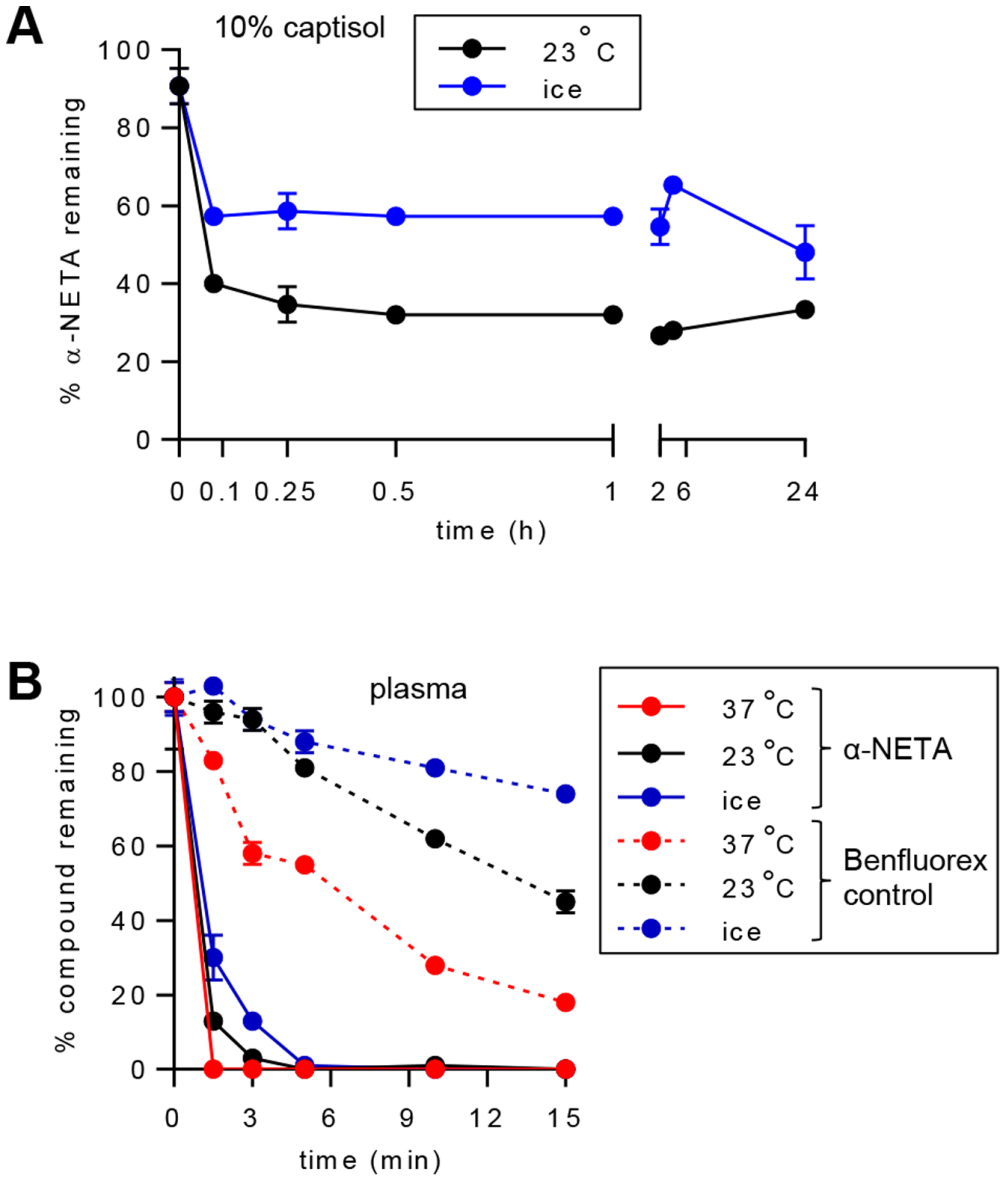
α-NETA stability. α-NETA was spiked into 10% captisol (A) or mouse plasma (B) and incubated at the indicated temperatures. At the indicated times, the samples were quenched and analyzed by mass spectrometry. The stability of Benfluorex (positive control compound) was also determined (B). Mean ± SD of triplicate wells for each point is displayed.

We next sought to determine the plasma pharmacokinetics of α-NETA. Based on its favorable drug formulation profile and its lack of action in the EAE model ([28] and our own observations), we selected the modified cyclodextrin captisol (10% solution in sterile water) as the formulation vehicle for α-NETA dosing *in vivo*. Following dissolution in 10% captisol, α-NETA rapidly (within 5 min) reacted or interacted with diluent or vessel components such that 40–60% of the parent compound (depending on the temperature of incubation) was no longer detectable by mass spectrometry (Fig. 2A). The remaining α-NETA in solution was, however, stable for at least 24 h (Fig. 2A). In initial *in vivo* dosing experiments, we injected α-NETA s.c. (10 mg per kg body weight, mg/kg) or i.v. (0.5 mg/kg) but were unable to detect any of the parent compound at any timepoint from 5–10 minutes through 24–48 hours post-injection (data not shown). To test its plasma stability *in vitro*, α-NETA (10 µM) was incubated in mouse plasma for various times at either 37°C, 23°C or chilled to 0°C, and then the samples were analyzed by mass spectrometry to quantify the percent of initial compound remaining. The well-characterized small molecule Benfluorex (1 µM) was treated in a similar manner as a positive control. Although the plasma stability of both compounds was dependent on temperature and the duration of incubation, α-NETA was significantly less stable than Benfluorex (Fig. 2B). At the earliest timepoint (90 s), α-NETA was undetectable in mouse plasma incubated at 37°C, and at 23°C there was just 13±2% of the initial amount remaining, while at 0°C there was 30±6% remaining (Fig. 2B). Following 3 minutes of incubation at 23°C there was just 3±1% of the initial amount remaining, while at 0°C there was 13±2% remaining. By 5 minutes there was essentially no detectable α-NETA at any temperature (Fig. 2B). Since α-NETA inhibits chemerin-dependent CMKLR1 signaling in the presence of serum proteins *in vitro* (Fig. 1 and data not shown), we conclude that α-NETA likely either forms rapid covalent bonds with plasma proteins while retaining its activity, or is rapidly converted to an active degradation product.

### Preliminary preclinical safety assessment of α-NETA

A common limiting toxicity for drug candidates is inhibition of the human Ether-a-go-go Related Gene (hERG) potassium channel, which regulates the repolarization phase of the cardiomyocyte action potential in the heart [29]. hERG inhibition causes cardic QT lengthening, which can lead to Torsade de Pointes, a rare but potentially fatal ventricular arrhythrmia [30]. α-NETA did not inhibit hERG channels at concentrations up to 92 µM [31] (Table 1), which satisfies a general rule-of-thumb requirement for drug development candidates for having an *in vitro* hERG IC_50_>10 µM [32].

Another important concern is the effect of compounds on cytochrome p450 (Cyp) enzymes, which are critical for detoxifying foreign chemicals and metabolizing drugs. Although there are more than 50 Cyp enzymes, Cyp1A2, Cyp2C9, Cyp2C19, Cyp2D6, Cyp3A4, and Cyp3A5 metabolize 90% of drugs [33]. α-NETA had weak-to-no inhibitory activity against Cyp2C9, Cyp2C19, and Cyp2D6 (IC_50_ ≧10 µM); moderate-to-weak inhibitory activity against Cyp1A2 (IC_50_: 8 µM); and potent but partial inhibitory activity against Cyp3A4 (IC_50_: 0.2 µM, 46% maximal inhibition at 57 µM) [34] (Table 1). Additional detailed *in vitro* studies using liver and intestinal microsomes with Cyp enzymes present in their native environments would be necessary to define potential time-dependent (mechanism-based) inhibition of Cyp3A4 and Cyp1A2, which can be predictive of *in vivo* drug-drug interactions [35]. Cyp inhibition does not necessarily pose an insurmountable barrier to drug development, as over 35 FDA approved drugs as well as grapefruit juice are known Cyp3A4 inhibitors [36]. Their use in combination with other drugs, however, requires careful monitoring to ensure patient safety.

In additional *in vitro* studies α-NETA was not acutely toxic to splenocytes, CMKLR1/L1.2 cells, or CMKLR1/CHO cells cultured *in vitro* in the presence of the compound for 90 min at concentrations up to 30 µM, as determined propidium iodide and annexin V viability staining (not shown).

**Figure 3:**
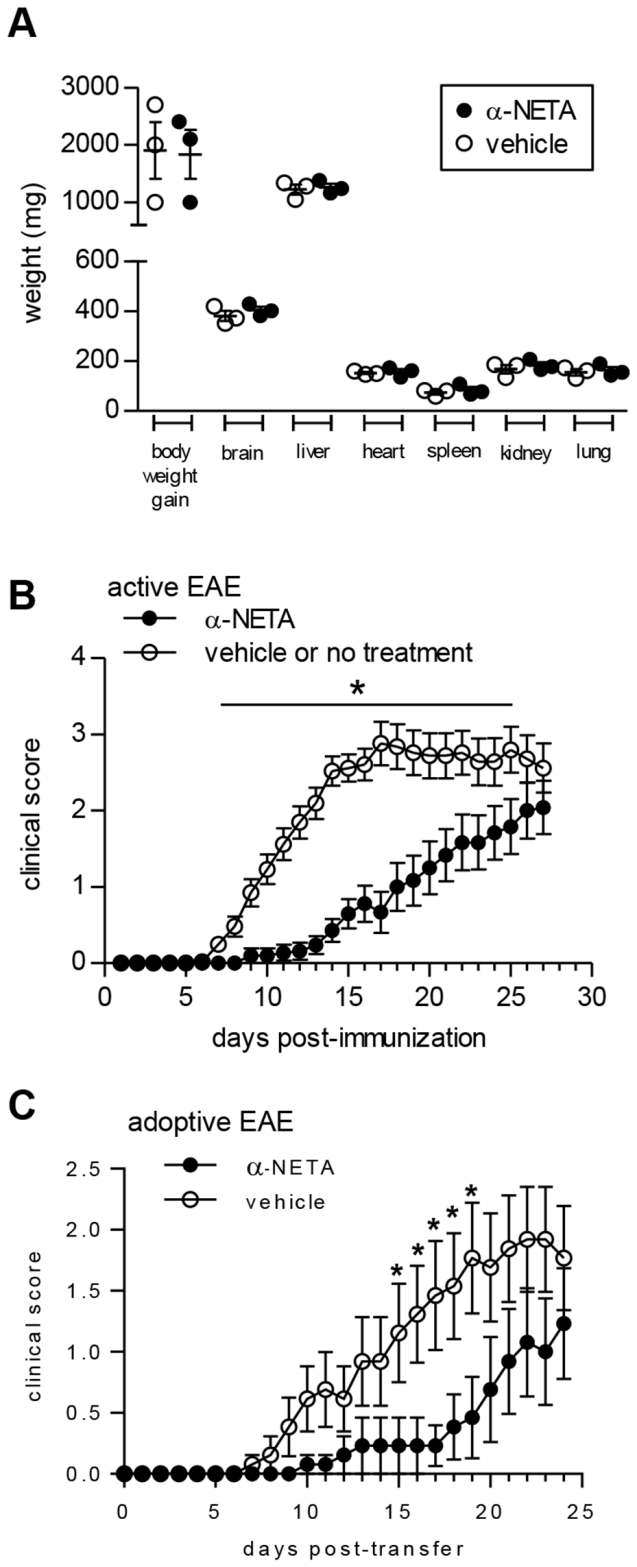
α-NETA is safe and suppresses clinical EAE. (A) C57BL/6 mice were injected s.c. with α-NETA (10 mg/kg) or vehicle daily for 16 days. The mice were then euthanized and vital organs weighed. Each symbol represents an individual mouse, and the bars indicate mean ± SEM, n = 3 mice per group. (B) EAE was induced in C57BL/6 mice by active immunization with MOG_35-55_/CFA. Mice received α-NETA (10 mg/kg daily, s.c. injection) beginning at the time of disease induction and were monitored daily for clinical disease as described in *Materials and Methods*. Control mice received either captisol vehicle or no treatment. The pooled data from five independent experiments with 7–10 mice per group is displayed, mean clinical score ± SEM. For days 1–15, n = 52 mice per group; for days 16–27, n = 24–25 mice per group. * *p*<0.05 by Mann-Whitney *U* test. (C) EAE was induced in C57BL/6 mice by passive transfer of MOG_35–55_-reactive lymphocytes derived from actively immunized EAE mice. Recipient mice received either vehicle or α-NETA (10 mg/kg) by daily s.c. injection beginning at the time of transfer and were scored daily for clinical disease. The pooled data from three independent experiments with 3–5 mice per group is displayed, mean clinical score ± SEM. * *p*<0.05 by Mann-Whitney *U* test.

As a preliminary assessment of potential *in vivo* drug toxicity, C57BL/6 mice were injected s.c. with various doses of α-NETA or vehicle daily for 16 days. α-NETA was well tolerated at doses up to at least 10 mg/kg per day: there were no differences in mouse survival, weight gain, appearance or general behavior; nor were there differences in the wet weight or macroscopic appearance (size, shape, coloration, structural features) of vital organs (Fig. 3A). Given the lack of adverse reactions *in vivo*, we next sought to test α-NETA for *in vivo* efficacy in the EAE model of MS.

### α-NETA suppresses clinical EAE

Trafficking molecules are key contributors to MS and EAE pathology, and our published data support a deleterious role for CMKLR1 in EAE. Given its ability to inhibit CMKLR1^+^ cell migration *in vitro*, we next asked if α-NETA could modulate immune pathology and inflammatory cell accumulation in the C57BL/6, MOG_35-55_-induced EAE model of MS. Beginning at the time of disease induction (day 0), we administered α-NETA daily to immunized mice via s.c. injection. Administration of α-NETA at doses as low as 3 mg/kg significantly delayed the onset of EAE (not shown), and higher doses of compound (10 mg/kg) completely suppressed clinical signs for an average of nine days beyond the first appearance of disease in control mice (Fig. 3B, Table 3). For mice that developed EAE, the average day of disease onset was day 10.6 post-immunization (p.i.) in control mice, compared to day 19.6 in mice dosed with 10 mg/kg α-NETA (Fig. 3B, Table 3). Nearly 30% of α-NETA-treated mice did not develop clinical EAE by day 27, compared with just 4% of control mice (Table 3). Furthermore, the severity of clinical disease was significantly reduced in the α-NETA-treated group through day 25 of the study (Fig. 3B). Long-term treatment with α-NETA, however, did not sustain suppression of disease, as clinical EAE in α-NETA-treated mice eventually became statistically indistinguishable from control mice (Fig. 3B). α -NETA also failed to reverse ongoing clinical EAE when treatment was initiated upon onset of clinical signs (data not shown).

**Table 3:** Clinical EAE in actively immunized mice treated with α-NETA^a^.

Treatment group	Mean day of onset (SEM)^b^	Incidence by day 27 post-immunization
vehicle	10.6 (0.7)	24/25 (96%)
α-NETA	19.6 (1.2)***	17/24 (71%)*

a Data are pooled from three independent experiments with each experiment consisting of 7–10 mice per group.

*p<0.05, as determined by Fisher's exact test;

***p<0.001, as determined by Mann-Whitney *U* test.

b Determined only for animals that developed EAE.

α-NETA significantly suppressed clinical signs of CNS autoimmune inflammatory disease in EAE induced by adoptive transfer of MOG-reactive T cells (Fig. 3C). Beginning at the time of adoptive transfer of encephalitogenic T cells (day 0), we administered α-NETA daily (10 mg/kg) via s.c. injection. α-NETA completely suppressed clinical signs for an average of six days beyond the first appearance of disease in control mice (Fig. 3C, Table 4). For mice that developed EAE, the average day of disease onset was day 13.0 post-transfer in control mice, compared to day 19.0 in mice dosed with 10 mg/kg α-NETA (Fig. 3C, Table 4). α-NETA reduced the incidence of EAE by day 24 by nearly 25%, although this did not reach statistical significance (Table 4). Furthermore, the severity of clinical disease was significantly reduced in the α-NETA-treated group from day 15–19 (Fig. 3C). Thus in two separate CNS autoimmune inflammatory disease models, α-NETA is efficacious in suppressing EAE.

**Table 4:** Clinical EAE in adoptive transfer model treated with α-NETA^a^.

Treatment group	Mean day of onset (SEM)^b^	Incidence by day 24 post-immunization
vehicle	13.0 (1.6)	9/13 (69%)
α-NETA	19.0 (2.0)*	6/13 (46%)

a Data are pooled from three independent experiments with each experiment consisting of 3–5 mice per group.

*p<0.05, as determined by Mann-Whitney *U* test.

b Determined only for animals that developed EAE.

### α-NETA suppresses histological EAE and alters leukocyte distribution in CNS and lymphoid tissues

**Figure 4:**
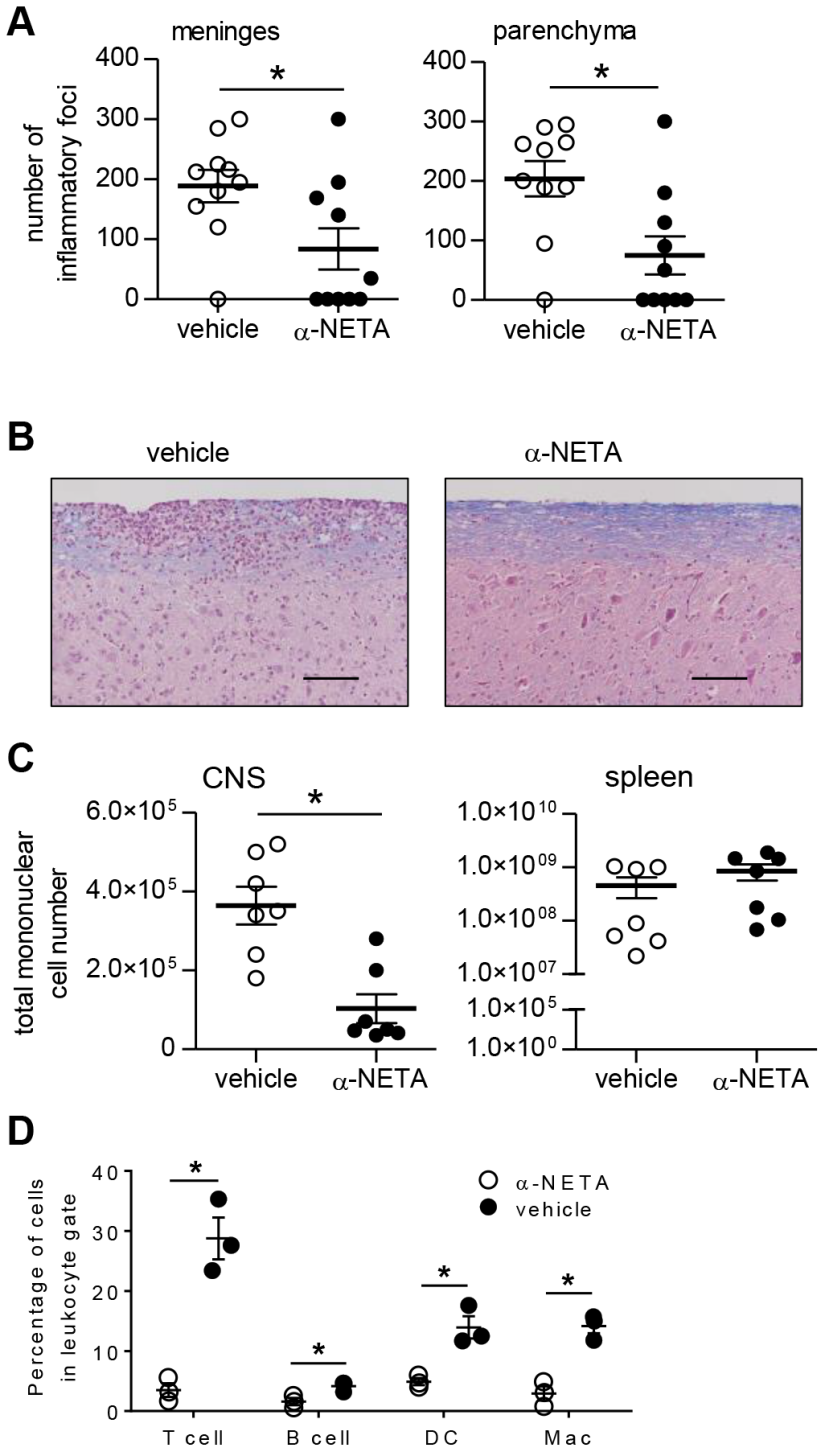
Reduced histological EAE in α-NETA-treated mice. (A) Brain and spinal cord tissues were harvested 16 days after active EAE induction from mice treated with α-NETA or vehicle control. Histological changes in meninges (*left panel*) and parenchyma (*right panel*) were evaluated as described in *Materials and Methods*. Results are pooled from two independent experiments with similar results, each with 4–6 mice per group. mean ± SEM, *p<0.05 as by Student's *t* test. (B) Representative spinal cord sections are shown from actively immunized vehicle- (*left panel*) or α-NETA- (*right panel*) treated mice that were euthanized at 16 days p.i. Meningeal and parenchymal mononuclear cell infiltrates typical of acute EAE in the spinal cord of a vehicle-treated mouse (*left panel*). Less meningeal and parenchymal infiltration, as well as reduced myelin loss (indicated by Luxol fast blue staining intensity), in the spinal cord of α-NETA-treated mice with EAE (*right panel*). Bar, 50 µm. (C) At day 16 p.i., mononuclear cells isolated from the CNS and spleen of EAE mice treated with α-NETA or vehicle control were enumerated by flow cytometry. n = 7 mice/group; mean ± SEM; *p<0.05 by Student's *t* test. (D) CNS mononuclear cells were isolated at day 16 p.i. from EAE mice treated with α-NETA or vehicle control. Cells were then stained with fluorophore-labeled monoclonal antibodies to identify the indicated leukocyte subsets: T cells (CD3+), B cells (B220+), dendritic cells (DC; CD3-B220-NK1.1-CD11c+) and macrophages (Mac; F4/80+CD11b+). *p<0.05 by Student's *t* test. Two independent experiments yielded similar results.

We assessed CNS tissues from mice treated with α-NETA or vehicle control for inflammatory cell infiltrates. Consistent with the clinical findings, mice treated with α-NETA in the active immunization model had fewer meningeal and parenchymal CNS inflammatory foci at the time of peak acute EAE (day 16 p.i.–Fig. 4A, B); however, there were no differences in numbers of CNS lesions between α-NETA and vehicle-treated mice that were evaluated after the development of chronic EAE (day 37 p.i. – data not shown). There was also qualitatively more intact myelin in the α-NETA treated group, as detected by increased light blue Luxol fast blue staining at the meningeal/parenchymal interface (Fig. 4B).

To determine whether α-NETA impacts inflammatory cell accumulation within effector and peripheral lymphoid tissues, we performed flow cytometric analysis on mononuclear cells isolated from the CNS tissues and spleens of mice with acute EAE from the active immunization model. At 16 days p.i., mice treated with α-NETA had fewer total cells in the CNS than vehicle-treated mice (p<0.05), consistent with the reduced clinical and histological EAE in these animals. In contrast, α-NETA-treated mice induced to develop EAE had more total cells in the spleen than their vehicle-treated counterparts; however, this difference did not reach statistical significance (Fig. 4C).

We also evaluated the distribution of effector leukocyte subsets in the CNS and periphery by flow cytometry. Treatment with α-NETA suppressed leukocyte accumulation, with no apparent preferential or selective effect on the distribution of T cells, B cells, dendritic cells, or macrophages within the CNS (Fig. 4D). NK cells express CMKLR1 [11], but treatment with α-NETA did not induce detectable changes in the total numbers of splenic NK cells (not shown). α-NETA treated mice, however, did have a significantly lower percentage of NK cells in the spleen (Table 5). Together, these data demonstrate that α-NETA limits leukocyte accumulation in the CNS, as well as alters leukocyte distribution in the periphery. Moreover, these results suggest that α-NETA may selectively modulate survival or migration of discrete leukocyte subsets.

**Table 5:** α-NETA limits accumulation of NK cells in the spleen during EAE^a^.

Treatment group	% of NK cells (CD3-NK1.1+) in lymphocyte scatter gate
vehicle	1.6 (0.1)
α-NETA	0.8 (0.1)*

a On day 16 post-EAE induction, spleen cells were harvested, stained, and analyzed by flow cytometry. n = 5 mice per group.

*p<0.05, as determined by Student's *t* test.

### *In vivo* α-NETA treatment does not alter lymphocyte proliferation and cytokine production

**Figure 5:**
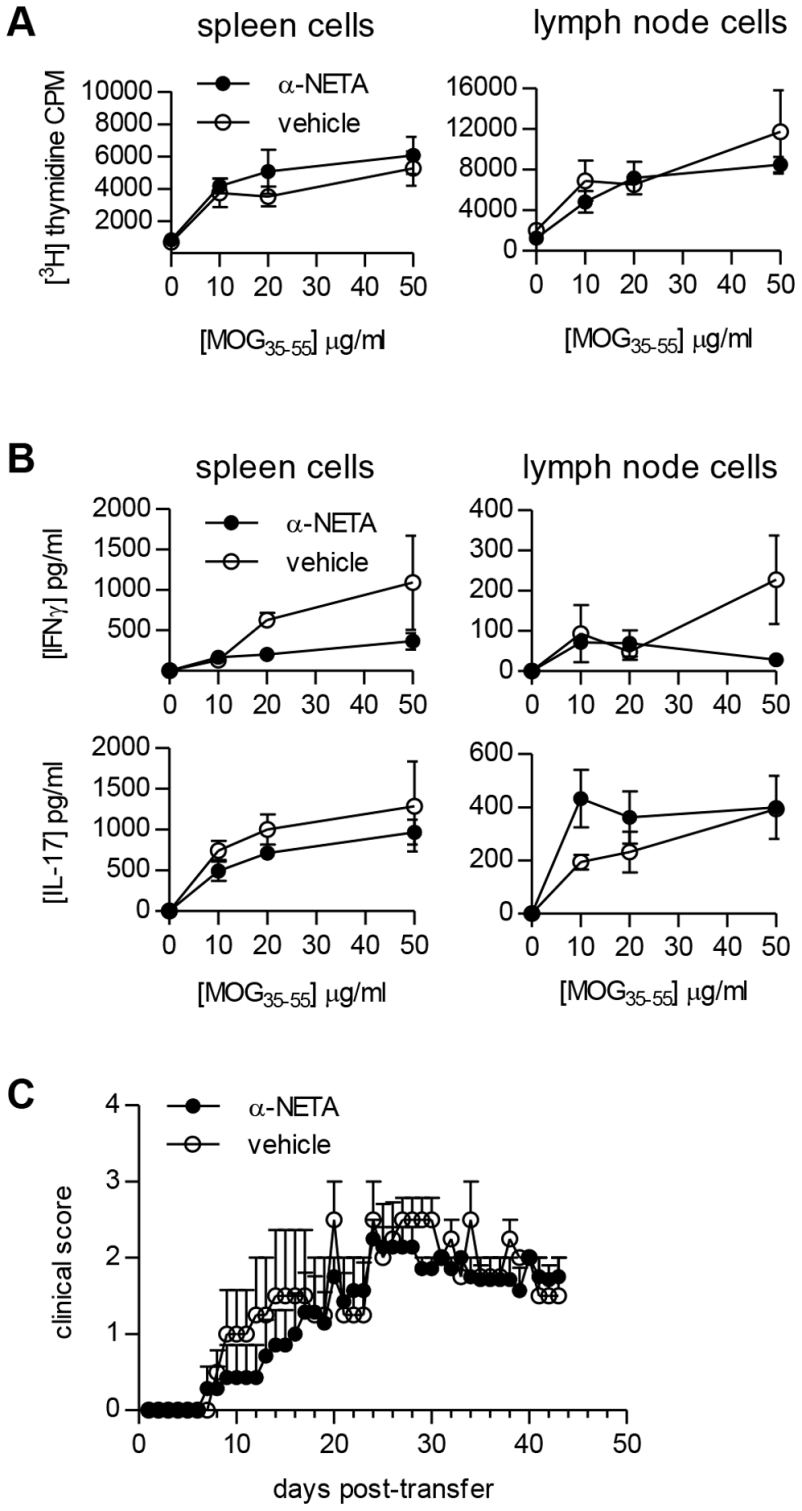
Recall proliferation and cytokine responses of lymphocytes from α-NETA-treated mice and induction of EAE by adoptive transfer of MOG-reactive lymphocytes. MOG_35–55_/CFA-immunized mice received α-NETA or captisol vehicle once daily. Ten days after immunization, spleen cells and draining LN cells were harvested and re-stimulated *in vitro* with the indicated concentrations of MOG_35–55_. After 72 h of stimulation, (A) proliferation and (B) cytokine production by spleen cells (*left panel*) or LN cells (*right panel*) were evaluated. Proliferation data are presented as mean cpm ± SEM (triplicate wells); ELISA data are presented as mean cytokine concentration ± SEM (triplicate wells). (C) EAE was induced in C57BL/6 mice by passive transfer of MOG_35–55_-reactive lymphocytes derived from vehicle-treated (n = 4) or α-NETA-treated mice (n = 7) as described in the *Materials and Methods*. The pooled data from two independent experiments is displayed, mean clinical score + SEM.

Controlling leukocyte migration is a primary function of chemoattractant receptors, and the reduced CNS leukocytic infiltration seen in α-NETA-treated mice with EAE supports a role for CMKLR1 in immune cell trafficking to neural tissues. Importantly, however, chemoattractant receptors can also regulate other cellular functions, such as survival and cytokine secretion [37]. Thus, we asked whether modulation of clinical EAE by α-NETA might be partly due to effects of the compound on lymphocyte activation *in vivo*. To assess antigen-specific recall proliferative and cytokine responses, we immunized mice with MOG_35-55_/CFA and administered α-NETA or captisol vehicle over a 10-day period. We then restimulated draining lymph node (LN) and spleen cells *in vitro* with MOG_35-55_ peptide. Overall, lymphocytes from α-NETA-treated mice proliferated and produced IFNγ and IL-17 at levels comparable to vehicle-treated mice (Fig. 5A, B). Thus, α-NETA treatment did not have noticeable effects on T cell recall proliferation or effector cytokine production, which is consistent with our published observations that CMKLR1 deficiency does not significantly impact lymphocyte responses in these assays [12]. Moreover, MOG-reactive lymphocytes from α-NETA-treated mice were fully capable of inducing EAE by passive transfer, further indicating that *in vivo* α-NETA administration does not appreciably inhibit T cell activation or CNS trafficking capability (Fig. 5C).

### α-NETA target selectivity *in vivo*

**Figure 6:**
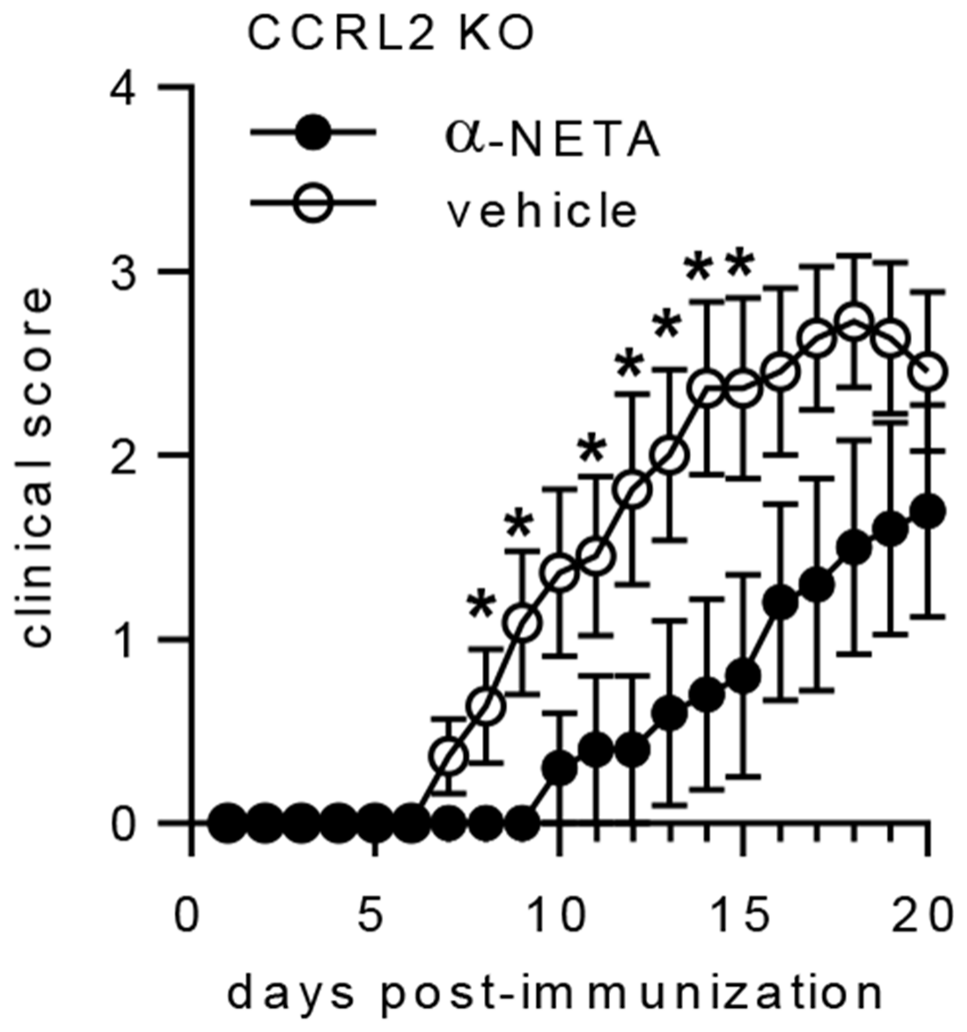
α-NETA suppresses clinical EAE in CCRL2 KO mice. (A) EAE was induced in CCRL2 KO mice by active immunization with MOG_35-55_/CFA. Mice received α-NETA (10 mg/kg daily, s.c. injection) beginning at the time of disease induction and were monitored daily for clinical disease. Control mice received either captisol vehicle or no treatment. The pooled data from two independent experiments with 5–6 mice per group is displayed, mean clinical score ± SEM. * *p*<0.05 by Mann-Whitney *U* test.

In addition to CMKLR1 and GPR1, there is a third chemerin receptor, chemokine (CC motif) receptor-like 2 (CCRL2), which serves to enrich local concentrations of chemerin [17], [38] but does not directly support chemerin-mediated intracellular calcium mobilization, chemotaxis [17], or β-arrestin2 recruitment (data not shown). Since α-NETA has at least some inhibitory activity against chemerin receptors CMKLR1 and GPR1 (Fig. 1B), we thought it prudent to ask if α-NETA could modulate the activity of CCRL2. However, the lack of classical signaling events makes it difficult to assess the potential action of antagonists against CCRL2. We hypothesized that if chemerin receptor CCRL2 was a bone fide target for α-NETA, then α-NETA would not effectively suppress EAE in CCRL2 KO mice *in vivo*. Beginning at the time of active disease induction (day 0), we administered α-NETA or vehicle control daily to immunized CCRL2 KO mice via s.c. injection. Vehicle-treated CCRL2 KO mice developed EAE with similar onset and clinical severity as WT mice (Fig. 3B, C). Administration of α-NETA (10 mg/kg) completely and significantly suppressed clinical signs for an average of four days beyond the first appearance of disease in control CCRL2 KO mice (Fig. 6, Table 6). For mice that developed EAE, the average day of disease onset was day 10.5 p.i. in control mice, compared to day 14.8 in mice dosed with 10 mg/kg α-NETA (Fig. 6, Table 6). While all CCRL2 KO control-treated mice developed EAE by day 20, 40% of α-NETA treated mice did not develop EAE (Table 6). Furthermore, the severity of clinical disease was significantly reduced in the α-NETA-treated group on days 8-15 (except for day 10) of the study (Fig. 6). Thus we conclude that α-NETA likely does not act through chemerin receptor CCRL2 to suppress EAE.

**Table 6:** Clinical EAE in actively immunized CCRL2 KO mice treated with α-NETA^a^.

Treatment group	Mean day of onset (SEM)^b^	Incidence by day 20 post-immunization
vehicle	10.5 (1.0)	11/11 (100%)
α-NETA	14.8 (1.2)*	6/10 (60%)†

a Data are pooled from two independent experiments with each experiment consisting of 5–6 mice per group.

b Determined only for animals that developed EAE.

*p<0.05 as determined by Students *t* test;

† p<0.05, as determined by Fisher's exact test.

Given the potential promiscuity of α-NETA (Table 1) for targets other than CMKLR1, we next asked if α-NETA had efficacy in CMKLR1 KO mice induced to develop EAE. However, this experiment is complicated by the fact that clinical EAE is significantly diminished in CMKLR1 KO mice compared to WT [12]. Nevertheless, beginning at the time of active disease induction (day 0), we administered α-NETA (10 mg/kg) daily to immunized CMKLR1 KO mice via s.c. injection. Although there was a slight delay in the mean day of onset between α-NETA treated vs. vehicle control-treated CMKLR1 KO mice, the difference was not significant (Table 7). There was a reduction in disease incidence by day 20 in α-NETA-treated CMKLR1 KO mice compared with vehicle-controls (40% vs. 80%, respectively), but this difference also was not statistically significant (Table 7). Thus, based on the aggregate of selectivity data we conclude that while α-NETA likely interacts with multiple targets *in vivo*, its inhibitory action against CMKLR1 is critical for maximal suppression of EAE.

**Table 7:** Clinical EAE in actively immunized CMKLR1 KO mice treated with α-NETA^a^.

Treatment Group	Mean day of onset (SEM)^b^	Incidence by day 20 post-immunization
vehicle	11.7 (1.0)	12/15 (80%)
α-NETA	14.3 (1.5)^ns^	6/15 (40%)^ns^

a Data are pooled from two independent experiments with each experiment consisting of 7–8 mice per group.

b Determined only for animals that developed EAE.

ns : not significant (p = 0.18) by Students *t* test for mean day of onset or by Fisher's exact test for day 20 incidence (p = 0.06).

## Discussion

Several features make CMKLR1 an attractive target for therapeutic manipulation in MS. For one, microglial cells and macrophages, key effector cells in MS, express this receptor. Also, given that clinical EAE is significantly reduced in CMKLR1 KO mice during the chronic phase of disease, CMKLR1 may be a target for progressive forms of MS, for which there are currently no approved therapies. Lastly, chemerin and CMKLR1^+^ plasmacytoid dendritic cells are present in the perivascular cuffs of MS patients, further demonstrating relevance for these molecules in the pathology of human disease. In this report, we identify α-NETA as a novel small molecule antagonist of CMKLR1. α-NETA inhibits CMKLR1^+^ cell migration *in vitro*, as well as chemerin-stimulated β-arrestin2 association with CMKLR1. Prolonged parenteral administration of α-NETA *in vivo* was well-tolerated, significantly delayed onset of clinical EAE, and limited leukocyte accumulation in the CNS. The compound also altered leukocyte distribution in peripheral lymphoid organs.

These studies highlight the utility of applying whole-cell functional assays to identify novel GPCR antagonists for prospective therapeutic applications. While traditional *in vitro* screening assays for modulators of GPCR activity monitor direct ligand: receptor binding or relatively ubiquitous biochemical phenomena (e.g., cyclic AMP production or Ca^2+^ flux), the β-ARR2 recruitment assay platform is suitable for identifying agonists as well as antagonists of GPCR activation. Furthermore, the β-ARR2 assay is expected to produce a lower false positive rate than standard screening methods [39]. Notably, it is possible that compounds with sub-optimal properties in traditional assays may in fact prove to be promising lead candidate molecules when tested in the β-ARR2 assay.

α-NETA was originally identified as a ChAT inhibitor [18]. It remains to be determined, however, whether α-NETA exerts any of its effects in EAE through modulation of ChAT activity, or if α-NETA can influence other CMKLR1-independent pathways, such as vitamin A metabolism via ALDH1A1 inhibition. Since the screening assay that identified α-NETA as a potent ALDH1A1 antagonist (IC_50_: 0.04 µM) was performed using purified enzymes and substrates [22], it is unclear if α-NETA would exhibit similar target potency on whole cells, given that its charged nitrogen would hinder passage through the cell membrane. Interestingly, ALDH1A1 is important for the conversion of vitamin A to retinoic acid, and all-trans retinoic acid was reported to suppress EAE *in vivo* [40]. It is reasonable to hypothesize that an inhibitor of ALDH1A1 may therefore exacerbate EAE *in vivo*. Whether this is occurring contemporaneously with CMKLR1-inhibition in EAE mice treated with α-NETA remains an open question; however, a more selective CMKLR1 antagonist unburdened from interacting with extraneous enzymatic targets may have improved *in vivo* efficacy in EAE.

The delayed onset of clinical signs in mice treated with α-NETA lends further support to a critical role for CMKLR1 in the pathogenesis of EAE. Notably, treatment with α-NETA recapitulates other key features of the CMKLR1 KO phenotype seen in MOG-induced EAE. For example, MOG-induced recall lymphocyte proliferation and cytokine responses were virtually indistinguishable in α-NETA- and vehicle-treated mice. Similarly, we previously showed that CMKLR1 deficiency did not significantly impact MOG-specific recall proliferation or pro-inflammatory cytokine production. Also, *in vitro*-generated MOG-reactive lymphocytes derived from α-NETA- and vehicle-treated mice were equally capable of inducing EAE by adoptive transfer (Fig. 5C). Thus, our data to date suggest that CMKLR1 has insignificant or redundant roles in the generation of functional MOG-reactive lymphocytes. Notably, α-NETA limited the accumulation of NK cells in the spleens of mice with EAE. Depletion of NK cells *in vivo* with anti-NK1.1 Abs has been shown to ameliorate MOG-induced EAE [41]. The role of CMKLR1 in NK cell activity or survival during EAE is not clear, but we are currently evaluating whether α-NETA modulates NK cell effector functions or migration to chemerin.

Delivery of chemoattractant receptor antagonists to the CNS at adequate concentrations *in vivo* may be a key determinant of therapeutic efficacy [42]. As a quaternary ammonium-containing compound, α-NETA may not sufficiently cross the blood-brain barrier (BBB), a property of many small molecule drugs that have been developed for therapy of CNS inflammatory diseases [43]. This may partially explain the inability of long-term α-NETA treatment to sustain suppression of clinical EAE. We speculate that α-NETA acts primarily on immune cells in the periphery to prevent/delay trafficking/infiltration into the CNS. However, once inflammatory cells enter the CNS, the BBB may exclude α-NETA and therefore limit its ability to disrupt immune cell migration conducive to CNS inflammatory disease. Structural modifications of α-NETA may enhance access to the CNS [44], and we are currently evaluating compound analogs for increased BBB penetrability. Initial SAR data, however, indicate that the loss of a methyl group (and loss of positive charge) from the α-NETA amine reduced its CMKLR1 antagonist activity at least ten-fold. Thus it may be challenging to improve CNS penetrability while retaining CMKLR1-target potency, as both may be linked to the quaternary amine. We are also investigating the relative roles of CMKLR1 in inflammatory versus demyelinating events– something that our studies to date have not specifically addressed – as this has implications for the design and implementation of CMKLR1-directed therapies.

The lack of prolonged and complete disease suppression by α-NETA may initially seem inconsistent with the reduced clinical disease seen in CMKLR1 KO mice with chronic EAE. In addition to the likely restriction of α-NETA effects to the periphery, however, there may be important (potentially confounding) developmental differences in immune function between these models, since KO mice lack the protein throughout development and their entire lifespan, whereas α-NETA-treated, CMKLR1-competent mice experience relatively transient (and only partial) inhibition of CMKLR1 activity.

Chemoattractant receptors and other trafficking molecules represent rational therapeutic targets in MS. Tysabri, a monoclonal antibody directed against the adhesion molecule α4 integrin, reduces disease activity and improves clinical MS [45], [46]. Tysabri must be infused intravenously, however, and use of this drug is associated with development of progressive multifocal leukoencephalopathy, melanoma, and a host of other ailments in some patients [2], [3], [4], [5]. Small molecule chemoattractant receptor antagonists offer many advantages over monoclonal antibodies and other biologics, including the potential for oral formulation and reduced immunogenicity. Use of small molecule drugs, however, does not come without risks. A prime example is Gilenya (also known as FTY720, or fingolimod), a modulator of the sphingosine-1-phosphate (S1P) receptor [47]. Gilenya improves relapse rates in MS patients [7], [48], but it has also been linked with serious side effects, including opportunistic infections and liver toxicity [6], [7].

The shortcomings of Tysabri and Gilenya underscore some of the potential pitfalls associated with inhibiting trafficking molecules for treatment of MS. Although they are a different class of therapeutic agent (biologic versus small molecule), Tysabri and Gilenya target molecules that are employed by virtually all leukocytes during physiological and pathophysiological circulation and trafficking to tissues. In contrast, CMKLR1 has a more restricted immune cell expression profile than α4 integrin and the S1P receptor, and CMKLR1 may in fact be selectively upregulated by discrete leukocyte subsets during pathogenic CNS inflammation. Thus, we predict that CMKLR1 inhibition will not induce a state of prolonged, systemic immunosuppression. There is, however, mounting evidence that the outcome(s) of chemerin: CMKLR1 interactions – i.e., pro-inflammatory or anti-inflammatory – can vary depending on the tissue and model under study [49], [50], [51], [52]. In addition, CMKLR1 and chemerin have emerging roles in adipose development, muscle cell differentiation, and metabolism [53], [54]. Our data suggest that targeting CMKLR1 during MS may ameliorate disease, but the potential effects of this approach on broader immune system and other functions must also be taken into consideration.

In 2013 dimethyl fumarate (Tecfidera) was approved by the FDA as a first-line therapy for adults with relapsing MS, although its mechanism of action remains largely unknown [55]. α-NETA and dimethyl fumarate share certain features such as small size (242 Da and 144 Da, respectively) and simplicity in structure. Both are electrophilic compounds and likely rapidly react with nucleophilic biomolecules such as glutathione *in vivo*. This may explain why both compounds are undetectable just minutes after *in vivo* dosing. Dimethyl fumarate is rapidly hydrolyzed to monomethyl fumarate, which is more stable, is 30% bound by serum proteins, and is the likely active metabolite. Finally, both compounds suppressed EAE in preclinical studies (Fig. 3B, C and [56]). Using the drug development path of dimethyl fumarate as a guide, we are attempting to identify the active component of α-NETA and define its pharmacokinetic profile, a key next step in its preclinical development.

The complexity and heterogeneity of MS poses challenges for attaining desirable clinical outcomes by targeting a single chemoattractant receptor. Small molecule inhibitors of CCR2 have been shown to ameliorate clinical EAE, but small molecule antagonists of CCR2 have failed in MS clinical trials [57]. Our results to date also suggest the existence of CMKLR1-independent pathways to CNS inflammatory pathology, as CMKLR1 KO mice retain susceptibility to EAE. Therefore, MS treatments that inhibit CMKLR1, CCR2 or other candidate receptors as part of a ‘polypharmacologic’ approach may be more efficacious than targeting a single receptor [58]. On the other hand, sufficient *in vivo* inhibition of a single critical chemoattractant receptor may significantly attenuate disease [42]. In support of this approach, a small molecule antagonist of CCR9 has shown promise in clinical trials for Crohn's disease, a complex, heterogeneous inflammatory disorder of the digestive tract [59]. The data presented in this report further implicate CMKLR1 as a GPCR with important roles in CNS pathology; and also suggest that targeting CMKLR1 with small molecule inhibitors warrants investigation as a possible treatment strategy for certain forms of MS.
